# Specifics

**Published:** 1882-11

**Authors:** E. J. Lee


### SPECIFICS.

*Facilis descensus Averni,*
*Sed revocare gradum, superasque evadere ad auras,*
*Hic labor, hoc opus est.*

In the well-known lines which are quoted above, Virgil tersely portrays the ease with which one can "descend" to evil habits and, conversely, the great difficulty experienced in "retracing" one's steps and "escaping" to the upper world of good habits. While this weakness of humanity is forcibly and frequently exhibited by the incidents of every-day life, we know of no instance that illustrates it more clearly than the ease with which one can glide, almost imperceptibly to himself, into a habit of routine prescribing. Routine prescribing, having "favorite" remedies for diseases, in homœopathic practice is, indeed, a veritable *descent*, a coming down from a higher to a lower plane of practice. For the system of practice developed by Hahnemann gives the physician a specific for *any* possible combination of symptoms; and as diseases are simply combinations of symptoms, it gives a specific for any disease, and, moreover, a definite, fixed law is given for finding this specific. On the other hand, the routine or the specific method of practice offers few remedies for yet fewer diseases, and these remedies produce most barren results. To illustrate: Hahnemann's system offers the diligent physician, say one hundred remedies for the symptoms of intermittent fever; the specific method would admit one, possibly two. As the *results*, the cures of the two methods of prescribing differ about as widely as their therapeutic strength—it is needless to say more.

It would be useless for us to discuss at this time this question of so-called specifics. It has no defenders, though many followers, for those who are the strictest specificists in their practice are often its most virulent opponents—in public! The fallacy and the weakness of the method have been frequently and clearly exposed. If the method were practically successful, it might be tolerated, but being, as it is, both false in theory and negative, yea even injurious in practice, one can but totally condemn it.

The purpose of these few lines is to call attention to the rapid progress many homœopathists are making in this routine or specific method of prescribing. Routine prescribing consists in giving remedies for diseases, "because it has cured that disease," without any reference to the symptoms. For instance, giving Phosphorus for "pneumonia," or Belladonna for "scarlatina," is routine prescribing. These drugs may be the proper remedies for some cases of these diseases, but this fitness must be based upon the symptoms present, not upon a name. The homœopathist must prescribe for the *symptoms* of the case to be treated, not for the *name* of the disease which his diagnosis tells him is present. As the physician does this, he takes his place as an homœopathist or as an eclectic. The homœopathic law demands that the symptoms of each patient, at every prescription, be carefully gathered and thoroughly compared with the pathogenesis of the remedy to be administered. Nothing less than this will preserve the physician from routine practice and secure him the fullest measure of success. The specifist prescribes, most generally, upon the *ipse dixit* of some learned (perhaps!) authority. Some one reports so many cases of such and such a disease as cured by this, that or the other remedy, *without a single failure.* (As, for instance, Chian turpentine for cancer, whose popularity came and went with almost meteoric celerity.) All the geese then hasten to try that remedy. Result, *failure;* they then seek another remedy. Failure after failure does not teach them that its method is fallacious; they still march on, cackling and cackling over alleged successes. Geese may once have saved Rome, but the specimens we have among us to-day seldom does as much for the sick.

We give a few specimens of this specific prescribing, culled at random from homœopathic literature. The following is a remarkable example:

"Usnea Barbata in Headache.—In February we published an account of this remedy, which an *accidental* proving showed to be very useful in obstinate headaches; we had then what we considered a liberal supply on hand, but *orders came pouring* in at such a rate that we could have disposed of as *many quarts* as we had ounces in stock."

Judging from the great demand for this remedy (as indicated by the words we have italicized in above quotation), one would suppose that the "accidental proving" alluded to was a thorough, masterly pathogenesis of the drug, giving in detail all its peculiar symptoms and clearly showing why it was "useful in obstinate headaches."

Reference to this "accidental proving" shows nothing of the sort! The "proving" was this: A man ate a little bit of this moss, a headache followed; thinking this must have been caused by the moss, a tincture of it was made and several headaches "cured" by it. Is this "proving" a sufficient basis for a homœopathic prescription? We think not. Yet we read that "orders came pouring in!" For what purpose? Simply this: a remedy for "*headache*" was recommended; its use required no labor, no thought; hence the specific-hunters poured in their orders. Probably each of these physicians had tried a dozen or so remedies for "*headache,*" and as each of these had proved a failure, he was ready to try another. Usnea Barbata will have its day, and be superseded by another "very useful remedy in obstinate headache." Such is the history of empirical prescribing. Yet these *Bourbons* seem to learn nothing; they appear not to know that such methods have been in vogue for thousands of years, and are to-day being discarded by the most advanced allopathists.

Another example of this folly:

"CUCURBITA PEPO IN VOMITING OF PREGNANCY.—Mrs. S., æt. 22, enceinte with second child, morning sickness set in, in the fourth week. I prescribed Cucurbita pepo tinct., one drop to half a glass of water, teaspoonful every half hour; cured in half a day, none after. She has had no trouble since.

"I would recommend to the profession a trial of Cucurbita in the sickness attending pregnancy. It will prevent much suffering, and the good mothers will ever be thankful to you for such prompt relief from this dreadful suffering."

We would ask, why should the profession try Cucurbita in morning sickness? Because this physician recommends it after curing *one* case—accidentally? Thinking men will scarcely consider *that* sufficient ground for an empirical experiment. If this be not the reason for this recommendation, then we would ask: what were the conditions upon which this physician prescribed the Cucurbita? Did he give it because his patient was named "S.," or because she was "æt. 22," or because she was "enceinte with second child," or was "the fourth week" his key-note? As these are the only facts related in connection with the case, and as a homœopathist must prescribe upon facts, we presume these are peculiar symptoms of Cucurbita pepo. But, how like old-fashioned allopathy such recommendations read!

As yet another instance, we recall reading a note in a journal, asking the editor if he could not give his readers a short article on "Leucorrhœa," giving *two or three* remedies for its treatment. The writer added: Look at Eggert's book and quit in disgust! Why one should quit in disgust after looking at a valuable book, we do not exactly understand. But this request for *two or three remedies* for leucorrhœa gives us this whole question of specifics in a nut-shell. Specific remedies for special diseases, easy labor-saving methods, are the need of the hour in medicine as elsewhere. Give me a remedy for headache, one for leucorrhœa, one for gonorrhœa, etc., etc., such is the cry of the successors of Hahnemann, Bœnninghausen, Dunham, etc. Such men have little knowledge of Homœopathy, and, it would seem, less concern about the lives intrusted to their care.

Unfortunately, these empirical recommendations are not the exceptions, but the rule. They are to be found, not only in homœopathic journals, but also in the text-books published for our instruction and guidance. The old veterans of our school prescribed for symptoms, not for diseases. The essays and books they published exhibited the same strong features. The essays and the text-books of too many of the more modern homœopathists are woefully lacking in this respect, and are correspondingly worthless.

Notwithstanding the fact that these departures are so apparent, a writer recently had the hardihood to ask why "modern homœopaths do not realize such 'cure work' as did the pioneers?" *Reason:* the "pioneer" practiced Homœopathy; the "modern" practices eclecticism. They have found, *facilis descensus Averni.* E. J. L.

